# Advances and challenges in regenerative dentistry: A systematic review of calcium phosphate and silicate-based materials on human dental pulp stem cells^☆^

**DOI:** 10.1016/j.mtbio.2023.100815

**Published:** 2023-09-23

**Authors:** B. Christie, N. Musri, N. Djustiana, V. Takarini, N. Tuygunov, M.N. Zakaria, A. Cahyanto

**Affiliations:** a Faculty of Dentistry, Universitas Padjadjaran, Jalan Sekeloa Selatan 1, Bandung, 40134, Indonesia; b Department of Dental Materials Science and Technology, Faculty of Dentistry, Universitas Padjadjaran, Jalan Raya Bandung Sumedang Km 21, Jatinangor, 45363, Indonesia; c Oral Biomaterials Study Center, Faculty of Dentistry, Universitas Padjadjaran, Jalan Sekeloa Selatan 1, Bandung, 40134, Indonesia; d Functional Nano Powder University Center of Excellence (FiNder U CoE), Universitas Padjadjaran, Jalan Raya Bandung-Sumedang Km 21, Jatinangor, 45363, Indonesia; e Faculty of Dentistry, Universiti Malaya, Kuala Lumpur, 50603, Malaysia; f Department of Restorative Dentistry, Faculty of Dentistry, Universiti Malaya, Kuala Lumpur, 50603, Malaysia

**Keywords:** Calcium phosphate, Silicates, Regenerative medicine, Dental pulp, Stem cells

## Abstract

Conventional dentistry faces limitations in preserving tooth health due to the finite lifespan of restorative materials. Regenerative dentistry, utilizing stem cells and bioactive materials, offers a promising approach for regenerating dental tissues. Human dental pulp stem cells (hDPSCs) and bioactive materials like calcium phosphate (CaP) and silicate-based materials have shown potential for dental tissue regeneration. This systematic review aims to investigate the effects of CaP and silicate-based materials on hDPSCs through in vitro studies published since 2015. Following the PRISMA guidelines, a comprehensive search strategy was implemented in PubMed MedLine, Cochrane, and ScienceDirect databases. Eligibility criteria were established using the PICOS scheme. Data extraction and risk of bias (RoB) assessment were conducted, with the included studies assessed for bias using the Office of Health and Translation (OHAT) RoB tool. The research has been registered at OSF Registries. Ten in vitro studies met the eligibility criteria out of 1088 initial studies. Methodological heterogeneity and the use of self-synthesized biomaterials with limited generalizability were observed in the included study. The findings highlight the positive effect of CaP and silicate-based materials on hDPSCs viability, adhesion, migration, proliferation, and differentiation. While the overall RoB assessment indicated satisfactory credibility of the reviewed studies, the limited number of studies and methodological heterogeneity pose challenges for quantitative research. In conclusion, this systematic review provides valuable insights into the effects of CaP and silicate-based materials on hDPSCs. Further research is awaited to enhance our understanding and optimize regenerative dental treatments using bioactive materials and hDPSCs, which promise to improve patient outcomes.

## Highlights

• Dental pulp stem cells benefit from calcium phosphate and silicate-based materials, advancing regenerative dentistry.
• Calcium phosphate and silicate-based materials promote crucial cellular activities for dental tissue engineering.
• Optimal concentrations of bioactive materials are essential for influencing dental pulp stem cells in regenerative dentistry.
• Bioactive materials facilitate the transformation of dental pulp stem cells into odontoblasts for dentin regeneration.
• Ongoing research aims to refine treatment protocols and enhance long-term safety and effectiveness in dental care through bioactive materials and stem cells.

## Graphical abstract

**Figure:**
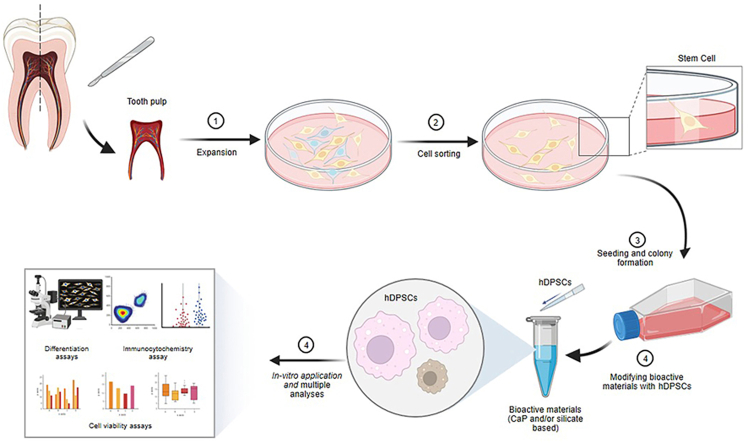

## 1 Introduction

Conventional dentistry plays a crucial role in preserving tooth health and function through various protective, restorative, and replacement measures. However, the finite lifespan of restorative materials presents limitations in maintaining long-term tooth health [1]. Regenerative dentistry has emerged as a potential solution to overcome these challenges and advance the field. Regenerative dentistry intends to rebuild dental tissues by leveraging the capability of stem cells and bioactive materials [[2], [3], [4]], offering a paradigm shift in dental treatments.

Stem cells are essential in dental tissue regeneration, able to differentiate, repair minor injuries, and regulate local inflammation [[5], [6], [7], [8], [9]]. Human dental pulp stem cells (hDPSCs) have shown remarkable regenerative potential and are a unique subset of mesenchymal stem cells found in teeth. These cells possess clonogenic capability and can differentiate into various stromal lineages, making them an ideal candidate for regenerative dentistry [[9], [10], [11], [12], [13]]. In vitro studies have demonstrated the differentiation of hDPSCs into dentin-pulp-like complexes, characterized by a mineralized matrix comprising vascularized fibrous tissues and odontoblastic cells. These distinctive characteristics position hDPSCs as a promising cell line for regenerative dentistry [3,4,8,11,14,15]. The hDPSCs are a suitable stem cell source for studying biological properties, suggesting their versatile suitability to different dentistry applications [[16], [17], [18], [19]].

**Fig. 1:**
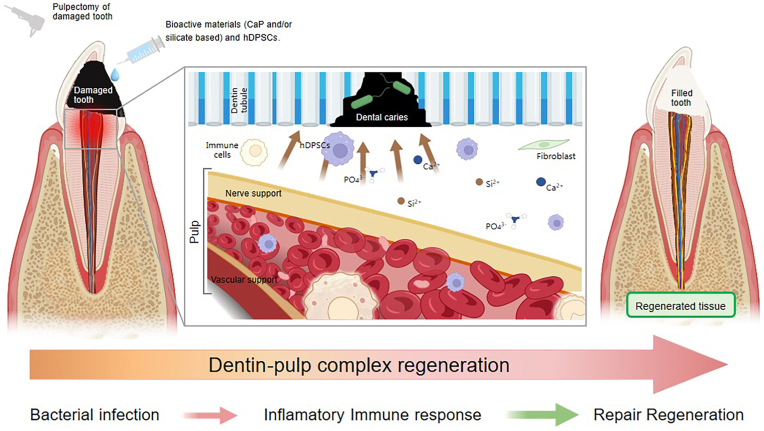
Regeneration of dentin pulp complex through incorporating bioactive materials (CaP and/or silicate-based) and hDPSCs in the damaged tooth.

Bioactive materials, including calcium phosphate (CaP) and silicate-based materials, are integral to regenerative dentistry, particularly in the field of endodontics. These materials interact with the surrounding environment and stimulate growth, promoting the regeneration of dental tissues. CaP, with its ability to mimic the mineralized phase of bone and teeth, exhibits desirable properties such as biocompatibility and the capacity to stimulate cell migration, proliferation, and differentiation [4,[20], [21], [22], [23], [24], [25]]. Silicate-based materials, known for their biocompatibility and ability to induce odontogenic and angiogenic behaviors, offer excellent sealing ability and antibacterial properties. Furthermore, sealers based on calcium silicate demonstrated superior cytocompatibility and more significant bioactive potential, resulting in increased formation of mineralized nodules, making them a recommended choice for clinical applications [[26], [27], [28], [29]]. Fig. 1 shows the mechanisms of bioactive materials combined with hDPSCs involved in dental pulp regeneration.

CaP has a long-standing history of applications in the medical and dental fields. It has various forms, such as cement, coating, scaffold, and paste, offering versatility [30]. CaP has the unique ability to mimic the mineralized phase of bone and teeth known as hydroxyapatite (HAp) [30,31]. CaP exhibits desirable properties such as biocompatibility, resorbability, load-bearing capacity, and injectability [30]. Furthermore, the surface characteristics of CaP have been shown to stimulate cell migration, proliferation, and differentiation, making it an excellent bioactive material for regenerative dental treatments [30,[32], [33], [34], [35], [36], [37], [38]].

Initially known as Portland cement, silicate-based materials were discovered in the late 1800s [39]. These materials have found primary use as endodontic sealers due to their extended working and setting time [40]. Like CaP, silicate-based materials demonstrate biocompatibility and can induce odontogenic and angiogenic behaviors while minimizing inflammation during the healing process [41,42]. Additionally, they exhibit excellent sealing ability, good marginal adaptation, and antibacterial properties [[43], [44], [45]]. These remarkable attributes position silicate-based materials as promising candidates for regenerative applications [39,40,[46], [47], [48]].

Based on these findings, a systematic literature review was conducted to address the research question: "What is the impact of CaP and silicate-based materials on the behavior and viability of hDPSCs as assessed through in vitro experiments, with a focus on potential therapeutic applications or limitations?". This study aims to provide a comprehensive synthesis of existing research to explain the precise effects of CaP and silicate-based materials on the behavior and viability of hDPSCs within controlled in vitro environments.

## 2 Methods

### 2.1 Study design and objective

Adhering to the Preferred Reporting Items for Systematic Review and Meta-Analyses (PRISMA) guidelines [49], this comprehensive systematic review aimed at examining the effect of CaP and silicate-based materials on dental pulp stem cells through in vitro studies published from 2015 onwards. Upon thorough scrutiny, ten eligible articles were identified and meticulously included in this systematic review. The selection procedure exclusively incorporated result-oriented research works that measured outcomes associated with activating dental pulp stem cells.

### 2.2 Search strategy

A comprehensive search strategy was conducted in PubMed MedLine, Cochrane, and ScienceDirect databases to identify relevant studies. For the investigation of CaP, the search query "((calcium phosphate [MeSH Terms]) AND dental pulp stem cells [MeSH Terms])) AND (in vitro [MeSH Terms])" was employed. Similarly, for the exploration of silicate-based materials, the query "((silicate-based materials [MeSH Terms]) AND dental pulp stem cells [MeSH Terms])) AND (in vitro [MeSH Terms])" was employed. The search was limited to studies published since 2015 to ensure the inclusion of recent research.

### 2.3 Eligibility criteria

Studies were selected based on the Participants-Intervention-Comparison-Outcome-Study Design (PICOS) scheme [50,51], as seen in Table 1. The inclusion criteria for study selection were as follows: in vitro studies published since 2015, studies focused on the effects of CaP or silicate-based materials, studies involving dental pulp stem cells as the target cell population, and studies reporting relevant outcomes related to the induction of dental pulp stem cells. Exclusion criteria included studies published before 2015, studies conducted in animal models, preclinical or clinical settings, and studies not reporting the specific effects of CaP or silicate-based materials on dental pulp stem cells.

**Table 1:** Inclusion and Exclusion criteria based on PICOS.

	Inclusion Criteria	Exclusion Criteria
Participants	hDPSCs	Other stem cells
Intervention	Addition of calcium phosphate or silicate-based materials	Other restoration materials
Comparison	A control group with no intervention or other group with different doses of the selected dental materials	Comparison to other stem cells
Outcome	Cellular activities of hDPSCs after the addition of calcium phosphate or silicate-based materials	Other impacts on hDPSCs after the addition of calcium phosphate or silicate-based materials
Study Design	In vitro studies published since 2015	In vitro studies published prior to 2015; other preclinical and clinical research

### 2.4 Screening and selection

**Fig. 2:**
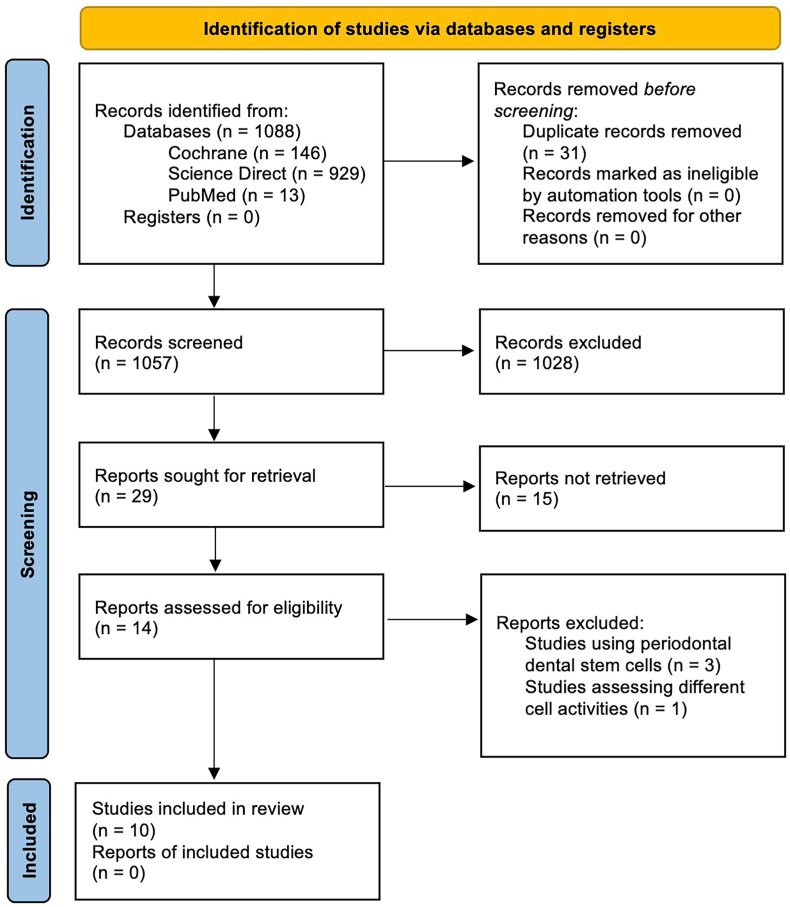
PRISMA flow diagram of the review and the study selection process.

The study selection process involved the application of a predefined inclusion and exclusion criteria framework based on the PICOS scheme mentioned earlier. These criteria were designed to specifically target studies that examined the effects of CaP and silicate-based materials on dental pulp stem cells in in vitro settings. A total of 1088 articles were initially screened, and through a meticulous evaluation, ten articles were found to meet the eligibility criteria and were subsequently included in this systematic review. The selection process was carried out independently by two reviewers (BC and NM), who carefully assessed the articles for eligibility based on the predefined criteria. Any discrepancies or disagreements were resolved through a third author (AC). Ten articles met the eligibility criteria and were included in this systematic review. The study selection process and the flow of article inclusion are presented in Fig. 2, following the PRISMA guidelines.

### 2.5 Data extraction

Two reviewers (BC and NM) conducted data extraction independently using a standardized approach. The extracted data encompassed various study characteristics such as author, publication year, and details on the bioactive materials on CaP and silicates. In addition, data on cell viability, adhesion, migration, proliferation, and differentiation were also collected. Outcomes related to the induction of dental pulp stem cells were also included. Any disagreements in data extraction were resolved through discussion and consensus between the reviewers.

### 2.6 Data items

The extracted data items encompassed various aspects of the included studies, including information on the study design, methodologies employed, qualitative outcomes, and relevant statistical analyses. The collected data provided a comprehensive overview of the effects of CaP and silicate-based materials on the induction of dental pulp stem cells in in vitro studies.

### 2.7 Registration and risk of bias (RoB) assessment

The complete protocol for this systematic review was registered in OSF Registries, and the registration DOI is www.doi.org/10.17605/OSF.IO/65Q7J or https://osf.io/65q7j/. Two reviewers (BC and NM) independently assessed the included study for RoB. The RoB assessment for the included studies was conducted using the Office of Health and Translation (OHAT) RoB tool [52], which evaluated five domains. The assessment aimed to critically appraise the quality and potential biases in the selected studies. By employing these comprehensive methods, we aimed to systematically gather and analyze relevant studies to investigate the effects of CaP and silicate-based materials on the induction of dental pulp stem cells.

## 3 Results

The initial search process yielded a substantial number of 1088 studies based on the chosen keywords. After careful evaluation, ten in vitro studies were considered eligible and included in this systematic review. These studies exhibited significant heterogeneity in terms of methodologies and materials, posing challenges for conducting meta-analyses or quantitative research.

A notable characteristic of the included studies was using self-synthesized biomaterials with innovative properties, deviating from commonly used commercial bioactive materials. While this approach showcases ingenuity, it also limits the generalizability of the findings to a broader population. Therefore, readers should exercise caution when interpreting and applying the results.

The findings from each study are summarized in Table 2, providing a comprehensive overview of critical details such as author, publication year, bioactive materials employed, methods used, and the resulting bio-cellular activities induced. These activities encompass dental pulp stem cell viability, adhesion, migration, proliferation, and differentiation.

**Table 2:** Summary of studies included in this systematic review.

Author, year	Bioactive Materials	Methods	Bio-cellular Activities Induction				
			Viability	Adhesion	Migration	Proliferation	Differentiation
AbdulQader et al. [33]	Calcium phosphate	HA and β-TCP phase: X-ray diffraction (XRD) Surface morphology: Field emission scanning electron microscopy (FESEM) Phosphate and calcium ions: calcium and phosphate colorimetric assay pH: pH meter Cell viability: MTT assay Alkaline phosphatase: ALP colorimetric assay Total RNA analysis and reverse transcription-polymerase chain reaction (RT-PCR) Statistics : One-way ANOVA and Tukey test using SPSS software	+	N/A	N/A	N/A	+
Chen et al. [53]	Mineral trioxide aggregate (MTA)	Cell and biomaterial morphology: scanning electron microscope (SEM) Proliferation: PrestoBlue assay Alkaline phosphatase: Sigma-Aldrich ALP Western blot: Invitrogen*,* densitometer*,* ImageJ software Mineralization: Alizarin Red S staining*,* optical microscope Wnt/β-catenin signaling: real-time polymerase chain reaction (PCR), RT-PCR Wnt/β-catenin effects towards odontogenic differentiation: cardamonin, *Alizarin Red S* staining Odontogenic activities: Enzyme-linked immunoassay (ELISA) Statistics: Scheffé multiple comparison testing	+	N/A	N/A	+	+
Tomás-Catalá et al. [54]	NeoMTA Plus, MTA Repair HP, and Biodentin	Si, P, Ca, and Sr ions: inductively coupled-mass spectrometry pH: pH meter Characterization of hDPSCs: flow cytometry Cytotoxicity: MTT assay Cell migration: wound-healing assay Adhesion: SEM Characterization of material: energy dispersive X-ray analysis	+	+	+	N/A	N/A
Pedano et al. [55]	Hydraulic calcium silicate cements (hCSCs) with *phosphopullulan* (PPL), Biodentine, Nex-Cem MTA, ZnO (negative control)	Characterization of hDPSCs: immunofluorescence staining Surface biomarkers: flow cytometry Cell colony: colony-forming assay Odonto/osteogenic differentiation: Stem-pro kit, staining Alizarin Red S Adipogenic differentiation: Cyagen, Oil Red O staining. Cell metabolism activity: Cell-cytotoxicity assay (colorimetric XTT) Cell proliferation: XTT assay Cell migration: wound-healing assay Odontogenic biomarker: RT-PCR Statistic: Non-parametric test Kruskal-Wallis	N/A	N/A	+	+	+
Wu et al. [56]	Tricalcium/dicalcium silicate-based composite, calcium hydroxide, tricalcium silicate	Cell viability*:* cell counting kit-8 (CCK-8) assay Alkaline phosphatase activity: ALP staining, semi-quantitative assay Gene expression: RT-PCR towards DSPP, OCN, and type-1 collagen (COL-1) Mineralization: Alizarin Red S staining Western blotting*:* BCA protein assay*,* incubated with DSPP, target protein detected with chemiluminescence Statistic: One-way ANOVA	+	N/A	N/A	+	+
Xia et al. [57]	Injectable calcium phosphate with gold nanoparticle (GNP-CPC), calcium phosphate cement	Characterization of GNP-CPC: SEM Mechanical characteristics: Universal Testing Machine (UTM) GNP-CPC phase: XRD analysis Degradation and gold ion: atomic absorption electroscopy Surface energy: water contact angle Protein adsorption: protein analysis kit Cell adhesion: CCK-8, Invitrogen, epifluorescence microscopy, Image-Pro Plus 6.0 Cell proliferation: CCK-8, microplate reader ALP activity: ALP assay kit, BCA protein assay kit Osteogenic gene expression: qRT-PCR Mineral synthesis: Alizarin Red S staining Gold nanoparticle absorption in hDPSCs: inductively coupled plasma optical emission spectrometry (ICP-OES) Statistic: ANOVA	N/A	+	N/A	+	+
Sun et al. [58]	Biodentine and iRoot Fast Set	Cell viability: live/dead assay Cell adhesion: SEM Cell migration: transwell assay Cell differentiation: qRT-PCR towards ALP, COL-1, OCN expression Statistic: ANOVA	+	+	+	N/A	+
Xia et al. [59]	Calcium phosphate with iron oxide nanoparticle (CPC-IONP)	Mechanical characteristics: UTM Surface contact angle: contact angle meter Protein adsorption: bovine serum albumin concentration Cell adhesion and proliferation: live/dead staining, Image-Pro Plus, CCK-8 Signaling: western blotting*,* RT-PCR ALP activity: ALP kit Osteogenic surface biomarkers: qRT-PCR towards ALP, COLI, OCN, WNT1, β-catenin, DKK.1, RUNX2, GAPDH Mineralization: live/dead assay staining Statistics: Kolmogorov-Smirnov test, ANOVA, and Bonferroni test	+	+	N/A	+	+
Li et al. [60]	Tricalcium silicate with zirconium oxide (TCS50), ProRoot MTA, TheraCal, ZnO	Cell viability: XTT assay Cell proliferation: XTT assay Cell migration: wound healing assay Odontogenic differentiation: RT-PCR Statistics: Kruskal-Wallis and Mann-Whitney U test	+	N/A	+,-	+,-	+
Mocquot et al. [61]	Bioactive glass (BAG) with large particles (LP) and fine particles (FP).	Cell metabolism: Alamar Blue assay ALP activity: K412-500 kit Cell adhesion and cytotoxicity: crystal violet staining, colorimetric assay (K329-1000) Cell viability: % cytotoxicity: [(OD DMSO – OD sample)/(OD DMSO] x 100 Cell morphology and spreading: confocal laser scanning microscopy (CLSM) Cell mineralization: Alizarin Red S staining Odontogenic expression: Rabbit Anti-Osteopontin Polyclonal Antibody staining, Alexa Fluor 555 Conjugated, monoclonal antibody, antibody SAB140275-100 G Statistic: ANOVA	+	+	N/A	N/A	+

**Notes:** +: biomaterial induced particular bio-cellular activity, -: biomaterial inhibited particular bio-cellular activity, N/A: particular bio-cellular activity was not tested in the study.

The analyzed studies focused on investigating the effects of various bioactive materials on bio-cellular activities in dental pulp tissue engineering. One commonly studied material was CaP, which demonstrated positive outcomes in multiple aspects. For instance, AbdulQader et al. (2015) examined cell viability, adhesion, proliferation, and differentiation in the presence of CaP [33]. Their findings indicated enhanced cell viability and differentiation, suggesting the potential of CaP for dental pulp tissue regeneration. Similarly, Xia et al. explored the impact of an injectable CaP composite on cellular activities [57]. They observed favorable outcomes regarding cell adhesion, proliferation, and mineral synthesis, further supporting the suitability of CaP for dental pulp tissue engineering.

Mineral trioxide aggregate (MTA) was another bioactive material widely investigated for its potential in dental pulp tissue regeneration. Chen et al. (2016) examined the effects of MTA on cell proliferation, alkaline phosphatase activity, and odontogenic differentiation [53]. Their results demonstrated positive outcomes in these areas, highlighting the potential of MTA as a suitable material for promoting cellular activities in dental pulp tissue engineering. Additionally, Sun et al. (2019) evaluated the impact of Biodentine and iRoot Fast Set, which are MTA-based materials, on cell viability, adhesion, migration, and differentiation [58,60]. Their findings revealed positive effects on cell viability, adhesion, and migration, further emphasizing the potential of MTA-based materials for dental pulp tissue engineering applications.

Hydraulic calcium silicate cements (hCSCs) were also included in the analysis. Pedano et al. investigated the effects of hCSCs with phosphopullulan on odonto/osteogenic differentiation, cell proliferation, migration, and colony formation [55]. Their results indicated positive outcomes regarding odonto/osteogenic differentiation and cell proliferation. These findings suggest the suitability of hCSCs as bioactive materials for promoting cellular activities in dental pulp tissue engineering.

The analyzed studies provide valuable insights into the bio-cellular activities induced by various bioactive materials in dental pulp tissue engineering. However, it is essential to note that the specific activities evaluated and the methods employed varied among the studies. CaP, MTA, and hCSCs demonstrated promising cell viability, adhesion, proliferation, and differentiation results. Further research and standardized evaluation methods are necessary to establish a comprehensive understanding of the bioactivity and effectiveness of these materials in dental pulp tissue engineering applications.

**Fig. 3:**
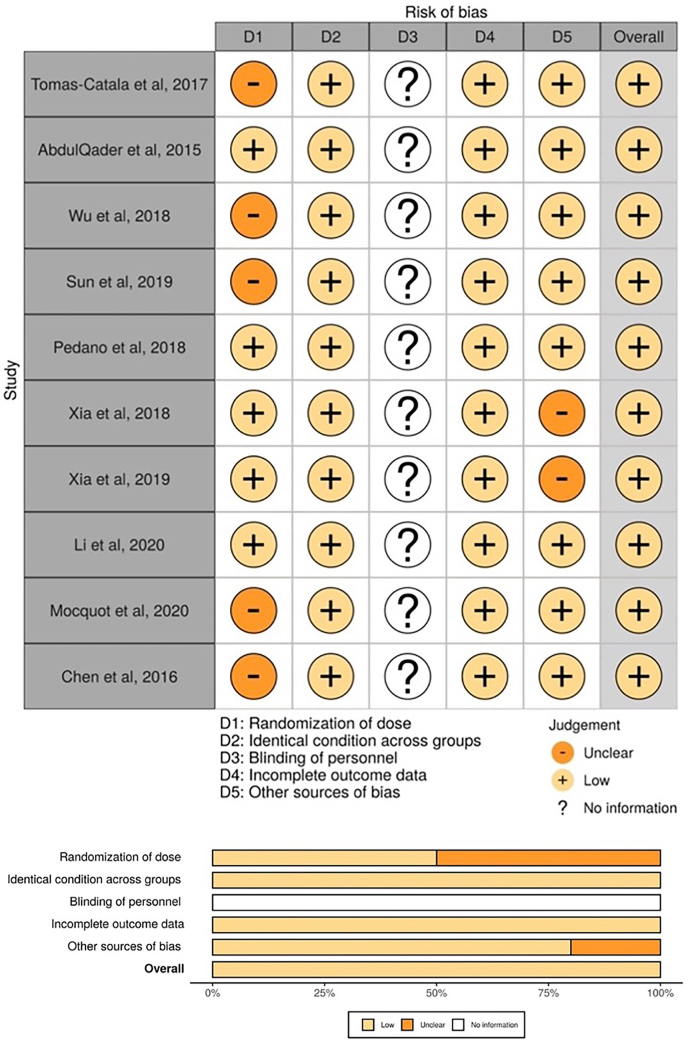
Risk of bias assessed using the Office of Health and Translation (OHAT) risk of bias assessment.

To assess the risk of bias in the selected studies, reviewers BC and NM employed the OHAT RoB tool. This tool considers various domains, including randomization, identical conditions, blinding, incomplete outcome data, and other potential biases. The overall bias assessment across all groups yielded satisfactory results, affirming the reliability and credibility of the reviewed in vitro studies as valuable sources of information. Visual representations in Fig. 3 have been included to enhance the clarity of the RoB findings. These figures offer concise and intuitive presentations of the outcomes of each included study, allowing for a deeper understanding of the RoB overall results.

Overall, the results of this systematic review provide valuable insights into the effects of CaP and silicate-based materials on dental pulp stem cells. Regardless of the heterogeneity of the included studies, the findings demonstrate promising outcomes in terms of dental pulp stem cell behavior and activity. These results support the potential utility of CaP and silicate-based materials in regenerative dentistry applications, further emphasizing the importance of continued research and exploration in this field.

## 4 Discussion

In the last two decades, CaP and silicate-based materials have found significant applications in dentistry [62,63]. Examples of these materials include ProRoot MTA [[64], [65], [66]], Biodentine [[67], [68], [69], [70], [71]], iRoot Fast Set [58,72], and BioRoot RCS [[73], [74], [75], [76]] which structure is almost similar to Portland cement [77,78]. One of the primary benefits of these materials lies in their capacity to stimulate remineralization, which is a mechanism closely aligned with the clinical attributes of the human oral cavity. When these materials come into direct contact with human tissues, they have the capability to release calcium ions that facilitate cell proliferation. Moreover, it establishes an antibacterial environment through its alkaline pH, thereby regulating cytokine production. Consequently, this promotes the migration and differentiation of cells responsible for generating hard tissues, forming hydroxyapatite on the cement surface, and establishing a biological seal [[79], [80], [81]]. To enhance the clinical characteristics of these materials, additional components such as modifiers as pharmaceutical substances are incorporated [[82], [83], [84], [85]].

Previous studies have provided evidence of cellular inducement activities by bioactive materials, and recent in vitro research included in this study further corroborates these findings. For instance, Chen et al. [53] analyzed the overexpression of WNT10a in hDPSCs, which promoted cell proliferation by increasing G2/M − and S-phase cells via the canonical WNT/β-catenin signaling transduction [53]. WNT signaling involves embryonic development, tissue homeostasis maintenance, and cellular activities. The canonical WNT/β-catenin pathway activated by the high stiffness of scaffolds induced odontogenic differentiation in hDPSCs [[86], [87], [88]]. Stem cells from the elderly, similar to cells from the young, exhibit a reaction to these signals [87,89]. In contrast, a study by Tiantian Wu et al. [56] demonstrated that the use of calcium silicate cement significantly influenced cell viability, the high alkalinity of the calcium silicate-based sealer media can up-regulate alkaline phosphatase activity and enhance mineralization [56,[90], [91], [92], [93], [94]].

AbdulQader et al. investigated the dose-dependency of hDPSCs viability when exposed to biphasic calcium phosphate (BCP) scaffolds, specifically three ratios of HAp and beta-tricalcium phosphate (β-TCP) [33]. The study revealed that BCP20, due to the increased release of alkalinity, calcium, and phosphate ions, decreased hDPSCs viability. Notably, BCP20 also induced the highest expression of bone sialoprotein (BSP), dental matrix protein-1 (DMP-1), and dentin sialophosphoprotein (DSPP), indicating its potential for dentin regeneration. A broader study by Tomás-Catalá et al. assessed various silicate-based materials, including NeoMTA Plus, MTA Repair HP, Biodentine, and MQ H2O [54]. The results demonstrated that these materials supported 95% viable hDPSCs expressing positive mesenchymal and hematopoietic markers. Other studies have also demonstrated positive effects on dental pulp regeneration through the utilization of calcium-phosphate-based scaffolds [[95], [96], [97], [98]]. However, in clinical applications, the use of a highly bioactive coating is often undesirable as it can potentially lead to instability of the implant or prosthesis. This is the main drawback of the bioglass formulations currently available on the market. Consequently, many surgeons tend to favor phosphate-based coatings due to their more favorable characteristics in this regard [99,100].

However, it is worth noting that several immunotoxic effects have been documented, and various toxic compounds emanating from dental materials can potentially to induce an inflammatory response in the surrounding tissue. While CaP and silicate-based materials exhibit biocompatibility, they remain foreign substances within the tissue. Inflammatory processes typically trigger the production of proinflammatory cytokines, which in turn often lead to a significant upregulation of COX-2 expression in various inflammatory disorders [101]. The study conducted by Hung et al. [102] reported immunocompatibility by assessing the expression of COX-2, along with the standard marker, during cellular inflammation. After three weeks of implantation, the expression of COX-2 was significantly elevated in both the MTA and calcium silicate groups when compared to the control group (p < 0.05). The acute inflammatory response could potentially be attributed to the dissolution of calcium oxide in the body's fluids, which likely increased the pH around the surrounding tissue. The initial inflammatory response to MTA is attributed to the elevated pH during the hydration process, which triggers the generation of inflammatory cytokines such as IL-1, IL-6, and COX-2, all contributing to inflammation [[103], [104], [105], [106], [107]].

Nonetheless, the expression of COX-2 decreased as the implantation time extended, eventually approaching levels similar to those observed in the control group after 12 weeks. Silicate-based materials induced a moderate reaction after one day, progressively decreasing over time [108]. An animal study revealed that Angelus MTA upregulated the adaptive immune response but had minimal or no impact on the production of pro- or anti-inflammatory cytokines [109,110]. Another study conducted by Rosanna et al. [16] underscores, that hDPSCs exposed to the three different bone substitute biomaterials such as OsteoBiol® GTO® [111], OsteoBiol® Gen-Os® [[112], [113], [114]], and OsteoBiol® Apatos® [115,116], promote both osteoblast and osteoclast activity. This finding highlights hDPSCs as a suitable stem cell source for investigating the effects of various biomaterials [16,19].

Cell adhesion plays a crucial role in the interactions between hDPSCs and biomaterials, making it an important aspect to investigate [57,58]. The biomaterials' biological, physical, and chemical properties can influence cell attachment. Yang Xia et al. conducted a study where gold nanoparticles were incorporated into injectable CPC, improving cell adhesion [57]. Gold nanoparticles offer numerous advantages, such as biocompatibility, large surface area, and easy preparation [[117], [118], [119], [120], [121]]. Through mechanical stress, gold nanoparticles can interact with proteins in the cytoplasm and extracellular matrix, subsequently triggering the activation of the p38/MAPK signaling pathway. This activation can potentially enhance the expression of mineralization genes [122].

Cell migration, a significant aspect studied in multiple research papers included in this review, demonstrated varied outcomes. Mariano Pedano et al. conducted an in vitro study examining widely available calcium silicate cement, phosphopullan (PPL), Biodentine, Nex-Cem MTA, and ZnO cement as the negative control [55]. The findings indicated that all silicate-based cement promoted cell migration, but the dosage influenced the results. The 100% diluted cement exhibited poorer cell migration compared to other concentrations. Notably, the experimental PPL cement demonstrated the highest cell migration among the groups, while Biodentine exhibited minimal cell migration across all concentrations. Numerous in vitro and in vivo studies have demonstrated that ions released from calcium silicate-based materials can facilitate cell attachment, proliferation, and differentiation and enhance the deposition of a mineralized matrix [55,[123], [124], [125], [126], [127]].

Biomaterials play a crucial role in influencing the cell proliferation of hDPSCs. A study by Xia Y et al. using iron oxide nanoparticle-calcium phosphate cement (CPC + IONP) demonstrated enhanced cell proliferation [59]. CPC has been found to influence the Wnt/β-catenin signaling pathway, subsequently affecting the differentiation of mesenchymal stem cells [59,128]. Several studies have also reported a notable impact of bioactive materials on cell proliferation [55,[129], [130], [131], [132]].

The differentiation of hDPSCs into odontoblasts is typically assessed through the expression of odontoblast markers such as DMP-1 and osteopontin. In an intriguing study by Caroline Mocquot et al. using an experimental silicate-based material revealed higher production and secretion of DMP-1 and osteopontin in the group with large particles compared to the group with fine particles. This difference could be attributed to the larger surface area and volume interacting with hDPSCs [61]. Additionally, odontoblast differentiation was found to be dose-dependent. Xin Li et al. investigated an experimental tricalcium silicate cement containing zirconium oxide and observed that a 25% diluted cement enhanced odontoblast differentiation without affecting hDPSCs viability, migration, and proliferation. Conversely, undiluted cement significantly inhibited the migration and proliferation of hDPSCs [60]. Furthermore, notable research trends included the predominant use of these materials for addressing dentine hypersensitivity [61,[133], [134], [135], [136], [137], [138], [139], [140]] or promoting dentine regeneration [65,141].

While the studies included in this review provide valuable insights into the cellular responses to bioactive materials, it is crucial to consider the limitations of these studies. Most of the research was conducted in vitro, and using hDPSCs as a model system may not fully replicate the complexity of dental pulp tissues in vivo. Additionally, variations in methodologies, experimental conditions, and sample sizes across the studies introduce some degree of variability and limit the generalizability of the findings. Further research is needed to explore the long-term effects and clinical outcomes associated with using these biomaterials. Despite these limitations, the reviewed studies provide valuable insights and lay the foundation for future investigations. Continued research efforts are necessary to refine our understanding of the interactions between hDPSCs and bioactive materials, paving the way for innovative approaches in dental tissue engineering and regenerative dentistry.

## 5 Conclusion

The studies reviewed in this analysis contribute to our understanding of the cellular responses of hDPSCs to various bioactive materials. These investigations have provided evidence of the inducement activities of these materials on hDPSCs, influencing vital aspects such as cell viability, adhesion, migration, proliferation, and differentiation into odontoblasts. The findings demonstrate the potential of bioactive materials to modulate the behavior of hDPSCs, with implications for dentin regeneration and tissue engineering applications. The modification of CaP and Silicate-based cements with hDPSCs holds great promise for enhancing both remineralization and pulp regeneration in dental treatments. This interdisciplinary approach combines materials science, regenerative medicine, and dentistry. Continued research and clinical trials are essential to fully harness the potential of this innovative approach.

## 6 Perspectives and future directions

The advancements in regenerative dental treatments hold great promise for overcoming the limitations of conventional dentistry and dental materials. These approaches aim to restore damaged teeth by regenerating the natural dental tissues rather than relying on foreign materials. Among the bioactive materials being explored, CaP and silicate-based materials have shown particular potential for regenerative treatments.

Studies have revealed that these materials can induce various cellular activities in hDPSCs, ranging from cell viability to differentiation into odontoblasts. Furthermore, recent findings have highlighted the specific relationship between bioactive materials and hDPSCs, including dose-dependent and time-dependent effects. Researchers have also made promising alterations to the biomaterials, enhancing their physical characteristics and promoting cellular activities in hDPSCs.

While there have been exciting developments in regenerative treatments using hDPSCs, significant research and development endeavors are necessary before these approaches can be implemented in clinical practice. Further studies are needed to enhance our understanding of bioactive materials and hDPSCs interactions, refine treatment protocols, and ensure these regenerative therapies' long-term safety and efficacy. With ongoing exploration and research in this field, these advancements are expected to revolutionize dentistry by providing regenerative solutions that restore natural dental tissues and enhance patient outcomes. Hence, due to these materials' pro-angiogenic, antimicrobial, and bioactivity properties, continuous research is being conducted to develop novel formulations [[142], [143], [144], [145], [146], [147], [148], [149]].

## Author contributions

Brenda Christie: Validation, Formal Analysis, Investigation, and Writing – Original Draft. Nabilla Musri: Validation, Formal Analysis, Investigation, and Writing – Original Draft. Nina Djustiana: Conceptualization, Data Curation, Writing – Review & Editing, and Supervision. Veni Takarini: Conceptualization, Data Curation, Writing – Review & Editing, and Supervision. Nozimjon Tuygunov: Formal Analysis, Investigation, and Visualization. Myrna Nurlatifah Zakaria: Conceptualization, Data Curation, Writing – Review and Editing, and Supervision. Arief Cahyanto: Conceptualization, Methodology, Data Curation, Writing – Review and editing, and Supervision.

## Declaration of competing interest

The authors declare that they have no known competing financial interests or personal relationships that could have appeared to influence the work reported in this paper.

## Data Availability

Data will be made available on request.
